# Development of a *Taq*Man-probe-based multiplex real-time PCR for the simultaneous detection of emerging and reemerging swine coronaviruses

**DOI:** 10.1080/21505594.2020.1771980

**Published:** 2020-06-03

**Authors:** Zhongzhou Pan, Jiaxuan Lu, Ningning Wang, Wan-Ting He, Letian Zhang, Wen Zhao, Shuo Su

**Affiliations:** MOE Joint International Research Laboratory of Animal Health and Food Safety, Engineering Laboratory of Animal Immunity of Jiangsu Province, College of Veterinary Medicine, Nanjing Agricultural University, Nanjing, China

**Keywords:** Real-time PCR, swine diarrhea virus, coronavirus, detection

## Abstract

With the outbreak of the recent severe acute respiratory syndrome coronavirus 2 (SARS-CoV-2) in 2019, coronaviruses have become a global research hotspot in the field of virology. Coronaviruses mainly cause respiratory and digestive tract diseases, several coronaviruses are responsible for porcine diarrhea, such as porcine epidemic diarrhea virus (PEDV), porcine deltacoronavirus (PDCoV), and emerging swine acute diarrhea syndrome coronavirus (SADS-CoV). Those viruses have caused huge economic losses and are considered as potential public health threats. Porcine torovirus (PToV) and coronaviruses, sharing similar genomic structure and replication strategy, belong to the same order *Nidovirales*. Here, we developed a multiplex *Taq*Man-probe-based real-time PCR for the simultaneous detection of PEDV, PDCoV, PToV, and SADS-CoV for the first time. Specific primers and *Taq*Man fluorescent probes were designed targeting the ORF1a region of PDEV, PToV, and SADS-CoV and the ORF1b region of PDCoV. The method showed high sensitivity and specificity, with a detection limit of 1 × 10^2^ copies/μL for each pathogen. A total of 101 clinical swine samples with signs of diarrhea were analyzed using this method, and the result showed good consistency with conventional reverse transcription PCR (RT-PCR). This method improves the efficiency for surveillance of these emerging and reemerging swine enteric viruses and can help reduce economic losses to the pig industry, which also benefits animal and public health.

## Introduction

Coronaviruses, belonging to the family *Coronaviridae*, order *Nidovirales*, are single-stranded, positive-sense RNA viruses with the largest genome among known RNA viruses [1,2]. According to genetic and antigenic characteristics, coronaviruses can be divided into four genera: α-coronaviruses, β-coronaviruses, γ-coronaviruses, and δ-coronaviruses [3–5]. Coronaviruses can cause respiratory and gastrointestinal diseases in animals and humans [6]. Generally, α- and β-coronaviruses only infect mammals, while γ- and δ-coronaviruses mainly infect birds, but some of them can also infect mammals [4]. Of note, coronaviruses exhibit a pronounced propensity for interspecies transmission as illustrated by important emerging viruses in humans such as SARS-CoV and Middle East respiratory syndrome-related coronavirus (MERS-CoV), as well as the recent SARS-CoV-2 that is causing a major human pandemic [7,8,9].

Compared to many other species, pigs are in frequent contact with both humans and other animals such as pets, livestock and wild animals, and theoretically possess a greater chance to promote cross-species viral transmission. There are currently six coronaviruses that can infect pigs: PEDV, transmissible gastroenteritis virus (TGEV), porcine respiratory coronavirus (PRCV), porcine hemagglutinating encephalomyelitis virus (PHEV), SADS-CoV, and PDCoV [10]. With the exception of PRCV and PHEV, the remaining four can all cause severe diarrhea, dehydration, and death in pigs [3,11]. PEDV is an α-coronavirus that causes long-lasting and extremely harmful swine diarrhea worldwide. PEDV may have originated from bat coronavirus [12]. It has high genome variability and different degrees of virulence among different strains [13]. PEDV strains can be divided into genotype G1 and genotype G2 with high genetic diversity. Both genotypes can cause catastrophic herd harm, of which the G2 variant has spread rapidly worldwide since 2010 [14–17]. SADS-CoV is also an α-coronavirus and is regarded to share a relationship with rhinolophus bat coronavirus HKU2 [18]. It was first discovered in January, 2017 in Guangdong, China. Afterward, there were no other reemerging SADS-CoV strains detected until it appeared again in Guangdong, China causing devastating damage to the local pig industry in 2019 [11]. This suggests possible periodic outbreaks of SADS-CoV could be observed in years to come. PDCoV, a δ-coronavirus, was first detected in 2012 and caused PEDV-like signs in pigs [19–21,22]. Birds can be considered as the natural hosts of δ-coronaviruses. Based on their ability to spread across species, δ-coronaviruses may “jump” the species barrier and adapt to mammals [23]. It has been reported that δ-coronaviruses have also been detected in Asian leopard cats and Chinese ferret-badgers [10,24]. PToV was first discovered by Kroneman *et al*. in 1998 [2]. PToV was considered as a coronavirus for a long time before the ICTV (International Committee on Taxonomy of Viruses) classified it into family *Tobaniviridae*, order *Nidovirales*. PToV has a high positive rate in swine diarrhea samples [25]. However, it remains to be a potential pathogen in pigs [25–27]. It should be noted that frequent recombination events involving PToV have been discovered, some of which, especially those on the Hemagglutinin-esterases (HE) or Spike (S) genes, which encode proteins relating to attachment and invasion, could lead to changes in pathogenicity or host-specificity [28–32].

So far, PEDV, PDCoV, SADS-CoV, and the coronavirus-like virus PToV have caused huge economic losses to the pig industry worldwide. In addition, their cross-species transmission ability may pose a threat to public health. In order to monitor these four viruses more efficiently, it is extremely important to develop a fast, simple, and accurate method for the detection and differentiation of those viral swine diarrhea pathogens. In clinical testing, multiplex real-time PCR has excellent performance. It has a larger detection capacity with higher speed and lower labor costs. However, the main reason hindering the application of multiplex real-time PCR on pathogen detection is the difficulty in assay design. Here, we developed a multiplex *Taq*Man-probe-based real-time PCR method for these four emerging and reemerging swine enteric viruses.

## Experimental section

### Primers and probes

To ensure the detection performance of primers used in the multiplex real-time PCR method, all available sequences of PEDV, PDCoV, PToV, and SADS-CoV from GenBank were obtained and analyzed. The conserved region of ORF1a was chosen for designing primers and probes for PEDV, PToV, and SADS-CoV, and the ORF1b region was chosen for PDCoV. Four sets of primers and probes were designed using the Oligo 7 (Version 7.60) software (Table 1). Primers and probes were synthesized by Sangon Biotech (Shanghai) Co., Ltd. Primers were also used for the construction of plasmid standards.

**Table 1.** Primers and probes*.

Pathogens	Primers and Probes	Sequences (5ʹ end to 3ʹ end)	Length (bp)	Gene	Position
PEDV	PE-Detection(F)	CTCCCTTGAATTTGAGTTCG	85	ORF1a	3041–3125^b^
	PE-Detection(R)	ACCACCTGTAACCTTGATAC			
	PE-Detection(Probe)	FAM-TTACCAACAGCCTTATTAAGCAC-MGB			
PDCoV	PD-Detection(F)	AAAGCTTTCAAGACAATACCT	87	ORF1b	15,130–15,216^c^
	PD-Detection(R)	TACGACAAACTCCTGAAAGCA			
	PD-Detection(Probe)	Texas Red-TACGATACGACTGCATTGGCCTAC-BHQ2			
PToV	PT-Detection(F)	TCATCCACCCAGTTCAAAT	73	ORF1a	1024–1096^d^
	PT-Detection(R)	TGCACAATTCTCTCTCCAAAT			
	PT-Detection(Probe)	VIC-CCTCAG^a^TTTCG^a^AGATAG^a^ACC-BHQ1			
SADS-CoV	SA-Detection(F)	CATTTGCCGTTCTTGACCAT	95	ORF1a	5269–5363^e^
	SA-Detection(R)	AACCCAGCAATTGTTATCTGAA			
	SA-Detection(Probe)	Cy5-CAAGTGCACGCTTACCATCAACTACT-BHQ3			

^a^LNA, Locked Nucleic Acid.

^b^GenBank accession No. AF353511.1.

^c^GenBank accession No. JQ065042.2.

^d^GenBank accession No. NC_022787.1.

^e^GenBank accession No. MF167434.1.

*All primers and probes are protected by patent. For any commercial use please contact the corresponding author.

All primers for conventional RT-PCR in this study referred to former reports [33–42].

### Virus strains and field samples

Clinical samples collected during 2017–2019 were preserved at −80°C in our laboratory. Those samples were mainly from Henan, Jiangsu, Anhui and Guangdong provinces in China. PCR templates were DNA or cDNA, preserved at −20°C. All positive samples were identified by singleplex conventional RT-PCR in our laboratory and confirmed with DNA sequencing by Sangon Biotech (Shanghai) Co., Ltd.

### RNA extraction and reverse transcription

Intestinal tissues or feces samples were treated with 3 to 5 volumes of PBS, mixed by shaking or vortex and supernatant was collected after centrifuged at 12,000 × g at 4°C for 15 minutes. Nucleic acids were extracted using the RNApure Virus Kit (Beijing ComWin Biotech Co., Ltd.) following the manufacturer’s instructions. Reverse transcription was performed using the HiScript III RT SuperMix for qPCR (+gDNA wiper) Kit (Nanjing Vazyme biotechnology Co., Ltd.).

### Construction of plasmid standards

The target fragments of PEDV, PDCoV, PToV, and SADS-CoV were amplified separately via PCR using the cDNA obtained in the previous step with the Phanta Max Super-Fidelity DNA Polymerase (Nanjing Vazyme biotechnology Co., Ltd.) following the manufacturer’s instructions. Primers used in the amplification were the same as used in multiplex real-time PCR method. The PCR fragments were then cloned into the pMD18-T vector (Takara Biomedical Technology (Beijing) Co., Ltd.) through TA colony and confirmed by DNA sequencing.

The plasmid copy number was calculated and the plasmids were diluted from 1 × 10^7^ copies/μL to 1 × 10^1^ copies/μL. Singleplex real-time PCR was performed for each virus using the 10-fold diluted plasmids to generate standard curves, based on which the E value (amplification efficiency), R^2^ (correlation coefficient), and the standard equation were calculated.

### Reaction conditions of the singleplex real-time PCR

As shown in Table S1, the total volume of the singleplex real-time PCR reaction was 20 μL, consisting of 10 μL of 2× AceQ qPCR Probe Master Mix (AceQ qPCR Probe Master Mix kit, Nanjing Vazyme biotechnology Co., Ltd.), 0.4 μL of each forward and reverse primer (10 μM), 0.2 μL of *Taq*Man probe (10 μM), 2 μL of template, and the remaining volume of nuclease-free water.

Amplification was carried out on a Roche LightCycler® 96 Instrument (Roche Life Science) using the following program: 95°C for 600 s; 40 cycles of 95°C for 10 s, 55°C for 10 s, and 72°C for 20 s. Fluorescence signal was automatically collected at the end of each cycle.

### Reaction condition optimization for multiplex real-time PCR

The singleplex real-time PCR assays for PEDV, PDCoV, PToV, and SADS-CoV described above were multiplexed into one reaction system consisting of 2× AceQ qPCR Probe Master Mix (AceQ qPCR Probe Master Mix kit, Nanjing Vazyme biotechnology Co., Ltd.), primers and probes for all four viruses, and templates. The multiplex reaction system was then optimized using different volumes of primers (10 μM) and probes (10 μM), and the optimal volumes of templates were determined. In the optimization stage, the final concentration of primers and probes in the system ranged from 1200 nM to 2400 nM and 200 nM to 1000 nM, respectively. The plasmid standards containing 1 × 10^3^ copies/μL were chosen as templates. The same instrument and real-time PCR program were used as described above.

### Sensitivity of the multiplex real-time PCR assay

To determine the limit of detection (LOD) of the multiplex detection method, we performed real-time PCR reactions for each virus separately, using 10-fold serial dilutions of standard plasmid templates ranging from 1 × 10^7^ copies/μL to 1 × 10^1^ copies/μL. To confirm the detection limit, a multiplex real-time PCR was performed using plasmid templates of all four viruses at the concentration of the presumable detection limit with 23 replicates for each concentration. The lowest concentration that met the positive detection rate of 95% was considered as the reliable LOD.

### Specificity of the multiplex real-time PCR assay

To rule out potential false positives caused by other viruses that may present in the samples, positive samples for PEDV, PDCoV, PToV, SADS-CoV, TGEV, porcine kobuvirus (PKV), classical swine fever virus (CSFV), porcine sapelovirus (PSV), porcine teschenvirus (PTV), and porcine rotavirus (PoRV) were tested using the multiplex real-time PCR detection method. All the cDNA samples were previously synthesized and stored in our laboratory.

### Repeatability of the multiplex real-time PCR assay

The assay was repeated three times with a 7-days interval, using 10-fold dilutions of the standard plasmid of each pathogen ranging from 1 × 10^7^ copies/μL to the LOD with three replicates per reaction. Each template was a mixture of standard plasmid of four pathogens at the same concentration. The coefficient of variation (CV) of the Cq values of the samples at each concentration in the three experiments was calculated to estimate repeatability.

### Simulation of co-infection by combining same concentration of standard samples

Plasmid standards of two, three or four target pathogens at the same concentration were randomly chosen and mixed as templates and detected using our new method. Three concentrations (1 × 10^7^ copies/μL, LOD and 10 times the LOD) of the plasmid standards were tested.

### Simulation of co-infection by combining different concentrations of standard samples

To simulate actual co-infection events, we mixed the plasmid standards of the four target pathogens with one at 1 × 10^7^ copies/μL and the other three at the LOD and then detected the template mixture using our multiplex detection method.

### Clinical sample detection

We tested 45 newly-collected samples from pigs showing signs of diarrhea and 56 previously-confirmed positive samples (31 with PEDV only, 16 with PDCoV only, 3 with PToV only, 1 with SADS-CoV only, 4 with PEDV and PToV, and 1 with PEDV, PDCoV and PToV) using our multiplex detection method. The clinical performance of our established methods was evaluated by comparing the results with those of singleplex conventional RT-PCR. Positive samples detected by either method were then confirmed through DNA sequencing by Sangon Biotech (Shanghai) Co., Ltd.

## Results

### Preparation of primers, probes and plasmid standards

**Figure 1.**
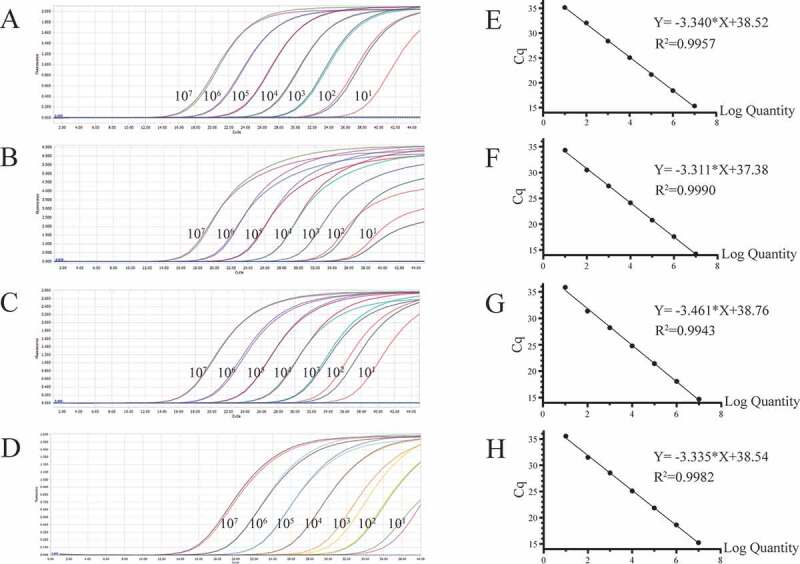
Preparation of plasmid standards. A-D: amplification curves (X-axis: Cycle, Y-axis: Fluorescence) of PEDV, PDCoV, PToV, and SADS-CoV for each plasmid standard of concentrations with 1 × 10^7^ copies/μL to 1 × 10^1^ copies/μL; E-H: standard curves of plasmid standards of PEDV, PDCoV, PToV, and SADS-CoV. All standard curves were conducted with software GraphPad Prism 8.

As shown in Table 1, the sequences of the primers and probes designed in this study are presented. PEDV, PDCoV, PToV, and SADS-CoV probes were labeled with FAM, VIC, Texas Red, and Cy5 separately. Plasmid standards with concentrations ranging from 1 × 10^7^ copies/μL to 1 × 10^1^ copies/μL of each pathogen were selected to perform a singleplex real-time PCR (Figure 1). The standard curves showed an acceptable amplification efficiency and correlation coefficient: PEDV R^2^ = 0.9957, E value = 99%; PDCoV R^2^ = 0.9990, E value = 100%; PToV R^2^ = 0.9943, E value = 94%; and SADS-CoV R^2^ = 0.9982, E value = 99%, indicating that our plasmid standards were qualified, and the primers and probes designed were efficient.

### Optimization of the multiplex reaction system

**Figure 2.**
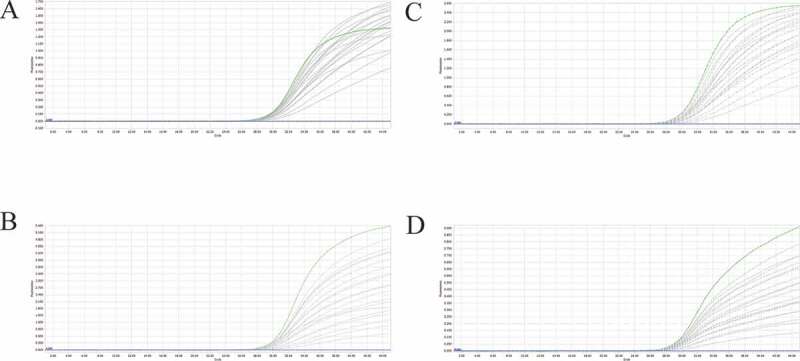
A-D: amplification curves (X-axis: Cycle, Y-axis: Fluorescence) of PEDV, PDCoV, PToV, and SADS-CoV detected by multiplex real-time PCR with different probe and primer concentrations. Plasmid standards with concentration of 1 × 10^3^ copies/μL were chosen as templates for the reactions. The four green lines are the amplification curves of four fluorescence of the most suitable reaction tube.

Among the four fluorophores used in the multiplex detection method, the fluorescence of Cy5 was the weakest and very susceptible to interference from other fluorophores due to its own physical properties. Therefore, the main purpose of the reaction optimization system was to improve the performance of the Cy5 fluorophores without hindering other fluorophores, and to achieve the best amplification efficiency (i.e. the lowest Cq value). We performed multiplex real-time PCR with final concentration of probes ranging from 200 nM to 1000 nM, and of primers ranging from 1200 nM to 2400 nM and compared the fluorescence intensity and Cq values of each possible combination. We concluded that the best final concentrations for probes and primers are 1000 nM and 2400 nM, respectively (Table 2 and Figure 2). The maximum volume of template that can be loaded in this system is 4.8 μL as shown in Table S2.

**Table 2.** Cq values of PEDV, PDCoV, PToV, and SADS-CoV detected by this multiplex real-time PCR assay with different probe and primer concentrations.

PEDV					PDCoV				
Probe concentration (nM)	Primer concentration (nM)				Probe concentration (nM)	Primer concentration (nM)			
	1200	1600	2000	2400		1200	1600	2000	2400
200	31.14	29.75	29.46	29.58	200	30.97	29.07	28.54	28.29
400	30.50	29.49	28.47	29.40	400	30.20	28.60	27.36	28.09
600	29.78	29.19	29.04	29.02	600	29.73	28.31	28.01	27.92
800	29.43	29.18	28.91	28.91	800	29.14	28.21	27.90	27.93
1000	29.55	29.08	29.11	28.84	1000	28.42	27.94	28.13	27.83
PToV					SADS-CoV				
Probe concentration (nM)	Primer concentration (nM)				Probe concentration (nM)	Primer concentration (nM)			
	1200	1600	2000	2400		1200	1600	2000	2400
200	31.63	29.35	29.48	29.24	200	29.82	29.10	29.08	28.99
400	30.87	29.57	28.04	28.77	400	30.56	29.51	29.26	29.35
600	30.17	29.05	28.81	28.72	600	31.47	29.77	29.68	29.51
800	29.49	28.73	28.65	28.40	800	31.66	30.14	29.42	29.59
1000	28.57	28.40	28.17	28.05	1000	33.66	30.58	30.21	29.96

### Sensitivity of the multiplex real-time PCR assay

**Figure 3.**
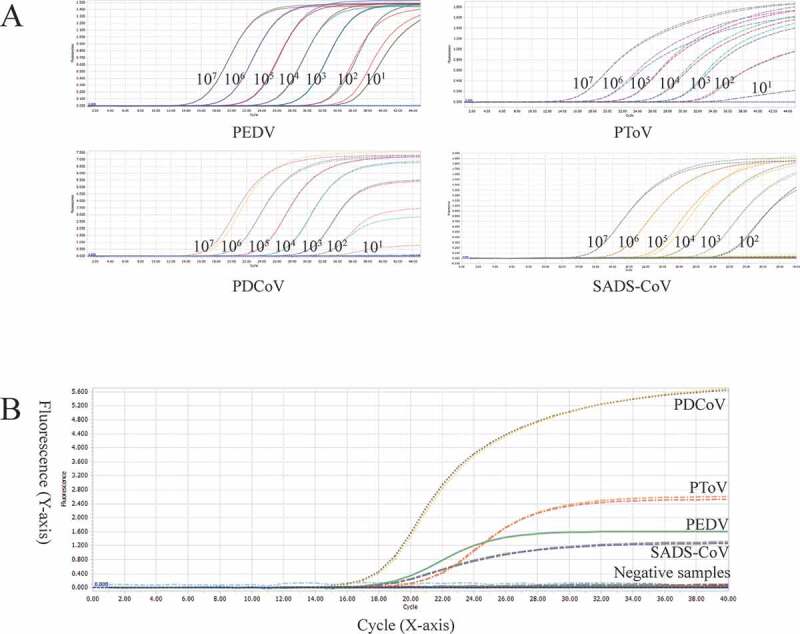
A: amplification curves (X-axis: Cycle, Y-axis: Fluorescence) of 10-fold serial dilutions (1 × 10^7^–1 × 10^1^ copies/μL) of plasmid standards of PEDV, PDCoV, PToV, and SADS-CoV detected by multiplex real-time PCR. B: four amplification curves represent samples positive for PEDV, PDCoV, PToV, and SADS-CoV detected by our multiplex real-time PCR assay; negative samples include TGEV, PoRV, PSV, PTV, CSFV, PKV, and negative control.

Using the optimized system and the plasmid standards of each pathogen with concentrations ranging from 1 × 10^7^ copies/μL to 1 × 10^1^ copies/μL, we found that the method could identify positive samples with the concentrations as low as 1 × 10^1^ copies/μL (Figure 3a and Table S3). However, follow-up experiments indicated that the detection rate of samples at 1 × 10^1^ copies/μL was less than 95% of replicates (Table 3). Thus, the reliable LOD of this method is 1 × 10^2^ copies/μL. In this experiment, the cutoff line of positivity was automatically decided by the LightCycler® 96 Instrument.

**Table 3.** Sensitivity of the multiplex real-time PCR assay.

Pathogens	Concentration	Total	Positive	Detection rate	95% detection rate
PEDV	1000 copies/μL	23	23	100.0%	> 95%
	100 copies/μL	23	23	100.0%	> 95%
	10 copies/μL	23	20	87.0%	< 95%
PDCoV	1000 copies/μL	23	23	100.0%	> 95%
	100 copies/μL	23	23	100.0%	> 95%
	10 copies/μL	23	14	60.9%	< 95%
PToV	1000 copies/μL	23	23	100.0%	> 95%
	100 copies/μL	23	23	100.0%	> 95%
	10 copies/μL	23	17	73.9%	< 95%
SADS-CoV	1000 copies/μL	23	23	100.0%	> 95%
	100 copies/μL	23	23	100.0%	> 95%
	10 copies/μL	23	12	52.2%	< 95%

The cutoff line of positivity is automatically decided by Roche LightCycler® 96 Instrument.

We set the cutoff line of positivity of our method at 35, which means samples with a Cq value less than or equal to 32 (≤ 32) are regarded as positive, higher than 32 but less than or equal to 35 (32 < and ≤ 35) are invalid, higher than 35 (>35) are negative. The criteria were set based on two reasons. First, the LOD of our detection method was 1 × 10^2^ copies/μL, of which the Cq value was around 32. Second, some samples at 1 × 10^1^ copies/μL were detectable, however, the detection rate was unqualified, and the Cq values of those detectable samples were around 35. All following experiments complied with these criteria.

### Specificity of the multiplex real-time PCR assay

The optimized method was used to detect PEDV, PDCoV, PToV, and SADS-CoV in positive samples and some other samples derived from pigs with diarrhea positive for TGEV, PKV, CSFV, PSV, PTV, and PoRV. As shown in Figure 3b and Table S4, the target pathogens were detected while the other pathogens were negative, indicating good specificity.

### Repeatability of the multiplex real-time PCR assay

As is shown in Table S5, most %CV values of the Cq values of the plasmid standard were less than 1% (81/96) with only a few %CV values ranging from 1% to 5% (15/96), indicating that this multiplex detection method is stable.

### Co-infection simulation experiment

**Figure 4.**
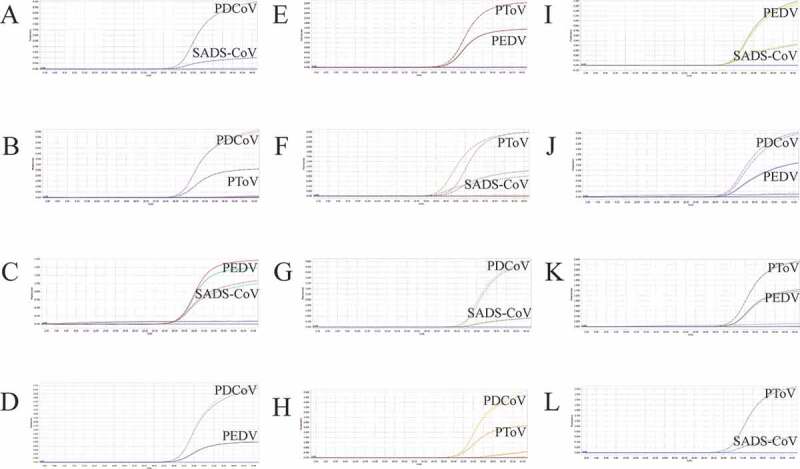
Co-infection simulation experiments with two pathogens. A-F: amplification curves (X-axis: Cycle, Y-axis: Fluorescence) of PDCoV + SADS-CoV, PDCoV + PToV, PEDV + SADS-CoV, PEDV + PDCoV, PEDV + PToV, PToV + SADS-CoV at concentrations of 1 × 10^3^ copies/μL; G-L: amplification curves of PDCoV + SADS-CoV, PDCoV + PToV, PEDV + SADS-CoV, PEDV + PDCoV, PEDV + PToV, PToV + SADS-CoV at concentrations of 1 × 10^2^ copies/μL. Two replicates were set per reaction.

**Figure 5.**
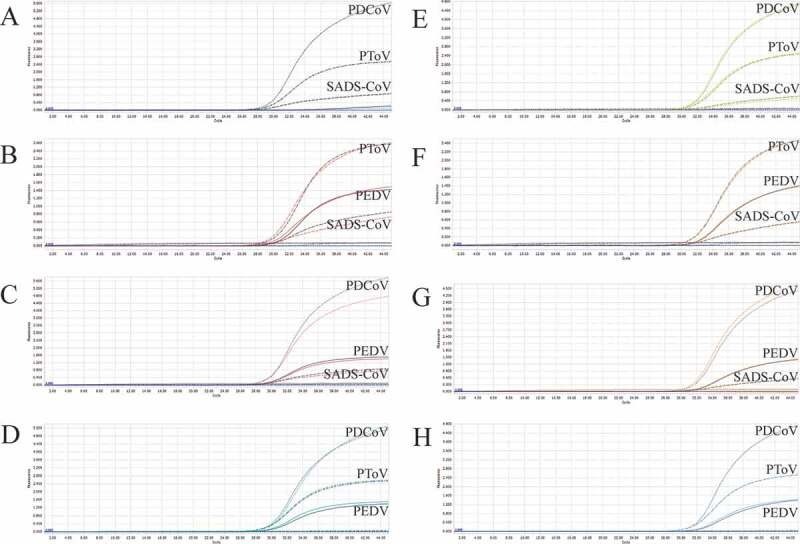
Co-infection simulation experiments with three pathogens. A-D: amplification curves (X-axis: Cycle, Y-axis: Fluorescence) of PDCoV + PToV + SADS-CoV, PEDV + PToV + SADS-CoV, PEDV + PDCoV + SADS-CoV, PEDV + PDCoV + PToV with concentration of 1 × 10^3^ copies/μL; E-H: amplification curves of PDCoV + PToV + SADS-CoV, PEDV + PToV + SADS-CoV, PEDV + PDCoV + SADS-CoV, PEDV + PDCoV + PToV with concentrations of 1 × 10^2^ copies/μL. Two replicates were set per reaction.

**Figure 6.**
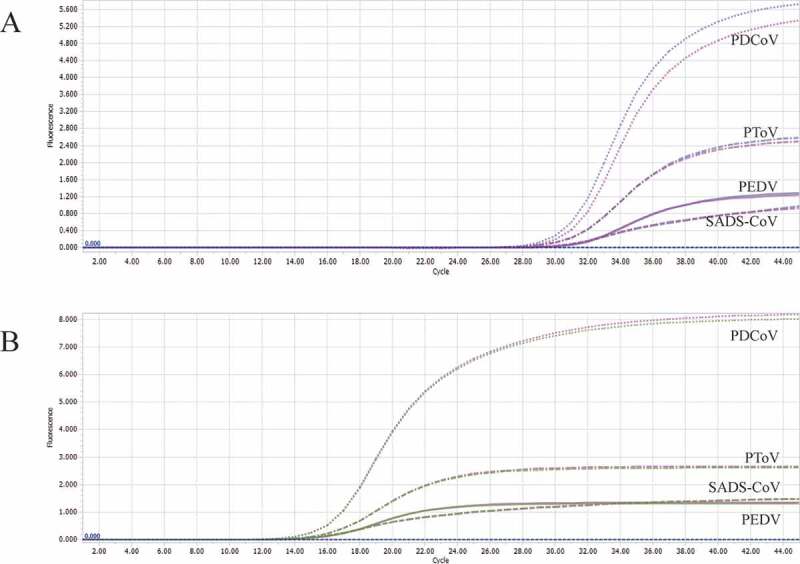
Co-infection simulation experiments with four pathogens. A-B: amplification curves (X-axis: Cycle, Y-axis: Fluorescence) of PEDV + PDCoV + PToV + SADS-CoV at concentrations of 1 × 10^2^ copies/μL and 1 × 10^7^ copies/μL. Two replicates were set per reaction.

**Figure 7.**
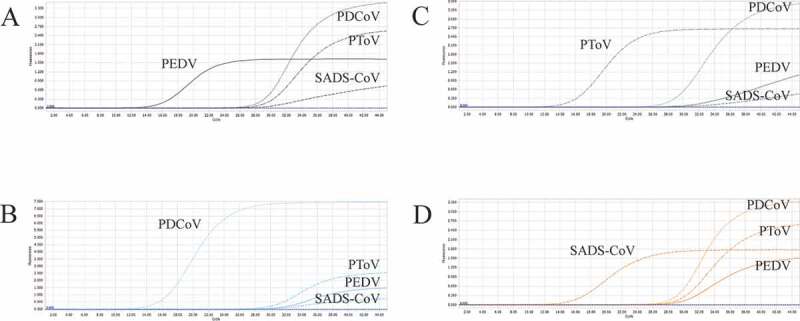
Co-infection of all the four pathogens at different concentrations. A: the concentration of plasmid standard of PEDV was 1 × 10^7^ copies/μL and the others were 1 × 10^2^ copies/μL; B: the concentration of plasmid standard of PDCoV was 1 × 10^7^ copies/μL and the others were 1 × 10^2^ copies/μL; C: the concentration of plasmid standard of PToV was 1 × 10^7^ copies/μL and the others were 1 × 10^2^ copies/μL; D: the concentration of plasmid standard of SADS-CoV was 1 × 10^7^ copies/μL and the others were 1 × 10^2^ copies/μL. Two replicates were set per reaction. X-axis: Cycle, Y-axis: Fluorescence.

We selected plasmid standards with concentrations of 1 × 10^7^ copies/μL, 1 × 10^3^ copies/μL, and 1 × 10^2^ copies/μL of different pathogens as templates to perform a co-infection simulation experiment. As shown in Figures 4, 5, and 6, the multiplex detection method could detect duplex, triplex, or quadruplex simulation co-infections of the target pathogens, even pathogens with different concentrations (Figure 7).

### Clinical sample detection

Two batches of clinical samples were tested by our established method to validate its performance in clinical use. The first batch consisted of 45 digestive tract samples recently collected from pig farms in China. The results of our method were 100% consistent with the results of singleplex conventional RT-PCR (Table S6). The second batch consisted of 56 positive samples stored in our laboratory, of which 31 were positive for PEDV alone, 16 were positive for PDCoV alone, 1 was positive for SADS-CoV alone, and 8 were positive for PToV (not necessarily alone). Our method was 100% consistent with singleplex conventional RT-PCR tests. It should be noted that among the 8 PToV positive samples, 3 samples were positive only for PToV, while the remaining 5 were also positive for other viruses, including 4 samples which were also positive for PEDV, and one positive for PEDV, PDCoV, and PToV (Table S6).

## Discussion

Virus cross-species transmission from wildlife reservoirs poses a remarkable threat to human and domestic animal health [18,43]. Coronaviruses can cross the species barrier and gradually adapt to new hosts [44,45]. For example, the recently emerged SARS-CoV-2 was estimated to have originated from bats spreading to wild animals or livestock and then to humans [46]. Animals may potentially serve as mixing vessels for the generation of novel recombinant coronaviruses and facilitate the viruses to expand their host tropism to humans [47]. In current swine breeding practices, humans have close contact with pigs, which further increases the possibility of viral transmission to humans, posing potential threat to human health [10]. Therefore, the detection of emerging and reemerging coronaviruses is of significance for farming and public health.

Among the four target pathogens in this study, PEDV is a coronavirus with considerably high prevalence and frequent recombination and rapid evolution rate [13]. SADS-CoV and PDCoV are two emerging coronaviruses and have been reported to be spreading and causing economic losses [11,23]. PToV is regarded as a swine diarrhea virus that has not caused huge economic losses [25]. However, given the high recombination rate and evolutionary characteristics of coronavirus and torovirus, we cannot underestimate their potential threat [28–30]. It is very likely that a virulent strain could emerge and cause unpredictable damage to the pig industry [13]. Thus, monitoring these currently less harmful viruses, such as SADS-CoV, PDCoV, and PToV, is of great significance.

Pathogen monitoring nowadays largely relies on laboratory detection methods. Singleplex conventional RT-PCR has been widely applied for pathogen detection [34–36,38]. However, such kind of assays are not convenient for the simultaneous detection of co-infection of multiple pathogens, which hinders further study of viral recombination events [3]. Given that co-infections of different coronaviruses and torovirus are common in the field and the high recombination frequency of coronavirus and torovirus, it is necessary to develop a fast, convenient detection method (e.g. multiplex real-time PCR) for the diagnosis of co-infections. PCR is fast, accurate and convenient for clinical sample screening. Real-time PCR is better than conventional PCR due to its faster, more sensitive and accurate detection capacity [48]. The use of probes is the most obvious and critical difference [49,50]. In general, conventional RT-PCR is less sensitive than real-time PCR. The LOD of our multiplex real-time PCR detection method can reach as low as 1 × 10^2^ copies/μL for each pathogen, while that of the singleplex conventional RT-PCR is generally around 1 × 10^3^ copies/μL to 1 × 10^4^ copies/μL [51]. Likewise, multiplex conventional RT-PCR shows no noticeable advantage in terms of sensitivity [34,52]. Hui *et al*. developed a multiplex conventional RT-PCR assay for pancoronaviruses, in which the limits of detection were no less than 1 × 10^3^ copies/μL [40]. Zhao *et al*. developed a multiplex RT-PCR detection for CSFV, porcine reproductive and respiratory syndrome virus (PRRSV), PEDV, and TGEV. The limit of detection of this method was 1 × 10^3^ copies/μL [52]. On the other hand, although multiplex real-time PCR combines high sensitivity and high detection efficiency, the design of qualified multiplex real-time PCR assays, especially of quadruplex quantitative real-time PCR assays, is challenging [48,49,53]. In multiplex real-time PCR assays, multiple sets of oligonucleotides exist simultaneously in the reaction system, increasing the possibility for nonspecific amplification, which poses high demands on the specificity of primers and probes.

Improvements in detection method can bring many benefits. Accurate detection of virus at lower concentration enables the diagnosis and prevention of porcine diarrhea at an early stage. Some virulent strains may cause severe signs at a low titer, and thus a sensitive detection method is indispensable in such situations. Due to its lower false negative rate caused by lower LODs, our new detection method enables more powerful surveillance over those four swine diarrhea viruses and, hopefully, can benefit the construction of pig farms with higher biosecurity. However, the improvement in sensitivity also increases the possibility of false positive results, which imposes higher requirements on the prevention of contamination during sampling and assay set up. Good practice in the laboratory is necessary in order to get credible results.

In short, an excellent and efficient detection method should accurately reflect the epidemiological data (e.g. the scale of the disease, the rate of transmission, and the severity of the epidemic), so as to be beneficial to the monitoring and prevention of the disease [53]. Multiplex real-time PCR achieves better detection capability in less time and with lower labor cost, but its development is still challenging compared to singleplex conventional RT-PCR due to technical difficulties at the development stage. To the best of our knowledge, this is the first multiplex real-time PCR detection method for PEDV, PDCoV, PToV, and emerging SADS-CoV, which often present simultaneously among pigs. The multiplex detection method developed here can detect multiple pathogens in a single reaction, making detection for co-infection more convenient. This method can ensure good specificity and sensitivity, which will undoubtedly save labor and material costs.

## Conclusion

Here, we developed a *Taq*Man-probe-based multiplex real-time PCR method for the simultaneous detection of emerging and reemerging PEDV, PDCoV, PToV and SADS-CoV of swine. The limit of detection can reach as low as 1 × 10^2^ copies/μL for each pathogen with good specificity and repeatability. The application of this method in clinical detection will not only improve the detection capacity, but also reduce workload and cost, benefiting clinicians and epidemiologists.

## Supplementary Material

Supplemental Material
