# Supplementary material for: Development of a TaqMan-probe-based multiplex real-time PCR for the simultaneous detection of emerging and reemerging swine coronaviruses
Source: Virulence. 2020 Jun 3;11(1):707–18. doi: 10.1080/21505594.2020.1771980 (PMC7549975; doi:10.1080/21505594.2020.1771980)
Supplement: Supplemental Material [file KVIR_A_1771980_SM0548.docx]

Development of a *Taq*Man-Probe-Based multiplex real-time PCR for the simultaneous detection of emerging and re-emerging swine coronaviruses

**Table S1** Reaction components of the singleplex real-time PCR assay (according to the instruction of AceQ qPCR Probe Master Mix kit).

| Components | Volume |
| --- | --- |
| qPCR SuperMix | 10 μL |
| Primer (F) (10 μM) | 0.4 μL |
| Primer (R) (10 μM) | 0.4 μL |
| Probe (10 μM) | 0.2 μL |
| Template | 2 μL |
| Nuclease-free water | 7 μL |

**Table S2** Reaction components of the multiplex real-time PCR assay.

| Components | Volume |
| --- | --- |
| qPCR SuperMix | 10 μL |
| Primer (F) (10 μM) | 0.6 μL for each |
| Primer (R) (10 μM) | 0.6 μL for each |
| Probe (10 μM) | 0.1 μL for each |
| Template + Nuclease-free water | 4.8 μL |

**Table S3** Cq values of 10-fold standard plasmid templates of each virus detected by multiplex real-time PCR.

| Log Quantity | PDCoV | |  | PToV | |  | PEDV | |  | SADS-CoV | |
| --- | --- | --- | --- | --- | --- | --- | --- | --- | --- | --- | --- |
|  | Replicate 1 | Replicate 2 |  | Replicate 1 | Replicate 2 |  | Replicate 1 | Replicate 2 |  | Replicate 1 | Replicate 2 |
| 7 | 13.98 | 13.88 |  | 14.76 | 14.87 |  | 14.98 | 15.06 |  | 15.26 | 15.41 |
| 6 | 17.30 | 17.32 |  | 18.69 | 18.64 |  | 18.16 | 18.12 |  | 18.96 | 18.85 |
| 5 | 20.49 | 21.26 |  | 22.03 | 21.95 |  | 21.41 | 21.53 |  | 22.76 | 22.25 |
| 4 | 23.82 | 23.88 |  | 25.36 | 25.52 |  | 24.73 | 24.81 |  | 25.71 | 25.66 |
| 3 | 27.64 | 27.17 |  | 28.75 | 28.61 |  | 27.98 | 27.97 |  | 29.23 | 29.14 |
| 2 | 30.28 | 30.48 |  | 31.91 | 32.24 |  | 31.73 | 31.80 |  | 32.41 | 32.41 |
| 1 | - | 35.36 |  | - | 34.11 |  | 35.34 | 34.34 |  | - | - |
| NC | - | - |  | - | - |  | - | - |  | - | - |

“NC” means taking the same volume of nuclease-free water as the template. The cut-off line of positivity is automatically decided by Roche LightCycler® 96 Instrument.

Based on the Cq values, we calculated the standard equation, R^2^ and E value of each virus with software GraphPad Prism 8: PDCoV: Y = -3.463*X+38.01, R^2^=0.9960, E=94.43%; PToV: Y = -3.264*X+38.17, R^2^=0.9957, E=102.48%; PEDV: Y = -3.329*X+38.17, R^2^=0.9995, E=99.71%; SADS-CoV: Y = -3.411*X+39.35, R^2^=0.9996, E=96.41%.

**Table S4** Specificity results of the multiplex real-time PCR.

| Positive samples | Singleplex conventional PCR detection and DNA sequencing^a^ | | | | | | | | | | Multiplex real-time PCR detection^b^ |
| --- | --- | --- | --- | --- | --- | --- | --- | --- | --- | --- | --- |
|  | PEDV | PDCoV | PToV | SADS-CoV | TGEV | PKV | PTV | CSFV | PSV | PoRV |  |
| PEDV | + | - | - | - | - | - | - | - | - | - | + |
| PDCoV | - | + | - | - | - | - | - | - | - | - | + |
| PToV | - | - | + | - | - | - | - | - | - | - | + |
| SADS-CoV | - | - | - | + | - | - | - | - | - | - | + |
| TGEV | - | - | - | - | + | - | - | - | - | - | - |
| PKV | - | - | - | - | - | + | - | - | - | - | - |
| PTV | - | - | - | - | - | - | + | - | - | - | - |
| CSFV | - | - | - | - | - | - | - | + | - | - | - |
| PSV | - | - | - | - | - | - | - | - | + | - | - |
| PoRV | - | - | - | - | - | - | - | - | - | + | - |

^a^ These samples were detected by singleplex conventional PCR detection and confirmed by DNA sequencing. ^b^ Criteria of positivity: Cq≤32, positive (+); 32<Cq≤35, invalid(?); Cq>35 or no singal detected, negative (-).

**Table S5** Repeatability results of the multiplex real-time PCR assay.

| PEDV | | | | | | | | | | | | | |
| --- | --- | --- | --- | --- | --- | --- | --- | --- | --- | --- | --- | --- | --- |
| Log Quantity | Group 1 | | | | Group 2 | | | | Group 3 | | | | CV % |
|  | rep1 | rep2 | rep3 | CV % | rep1 | rep2 | rep3 | CV % | rep1 | rep2 | rep3 | CV % |  |
| 7 | 14.82 | 14.77 | 14.89 | 0.41 | 14.88 | 14.71 | 14.83 | 0.59 | 14.97 | 14.85 | 14.92 | 0.40 | 0.53 |
| 6 | 18.13 | 18.18 | 18.18 | 0.16 | 18.20 | 18.11 | 18.13 | 0.26 | 18.16 | 18.22 | 18.20 | 0.17 | 0.21 |
| 5 | 21.42 | 21.46 | 21.60 | 0.44 | 21.50 | 21.43 | 21.52 | 0.22 | 21.57 | 21.62 | 21.58 | 0.12 | 0.35 |
| 4 | 24.71 | 24.74 | 24.70 | 0.08 | 24.89 | 24.83 | 24.92 | 0.18 | 24.73 | 24.92 | 24.79 | 0.39 | 0.36 |
| 3 | 27.82 | 27.98 | 28.09 | 0.49 | 28.01 | 28.04 | 28.02 | 0.05 | 27.64 | 27.93 | 27.86 | 0.54 | 0.50 |
| 2 | 30.85 | 31.02 | 30.47 | 0.91 | 29.64 | 30.79 | 30.80 | 2.19 | 30.19 | 30.25 | 30.31 | 0.20 | 1.42 |
| NC | - | - | - | - | - | - | - | - | - | - | - | - | - |
|  |  | | | | | | |  |  | | | | |
| PDCoV | | | | | | | | | | | | | |
| Log Quantity | Group 1 | | | | Group 2 | | | | Group 3 | | | | CV % |
|  | rep1 | rep2 | rep3 | CV % | rep1 | rep2 | rep3 | CV % | rep1 | rep2 | rep3 | CV % |  |
| 7 | 13.57 | 13.56 | 13.61 | 0.19 | 13.45 | 13.45 | 13.45 | 0.00 | 13.61 | 13.46 | 13.52 | 0.56 | 0.51 |
| 6 | 16.95 | 17.01 | 18.22 | 4.12 | 17.00 | 16.93 | 16.89 | 0.33 | 16.99 | 17.03 | 17.00 | 0.12 | 2.44 |
| 5 | 20.36 | 20.42 | 20.49 | 0.32 | 20.45 | 20.40 | 20.43 | 0.12 | 20.59 | 20.58 | 20.54 | 0.13 | 0.40 |
| 4 | 23.70 | 23.65 | 23.67 | 0.11 | 23.79 | 23.84 | 23.83 | 0.11 | 23.72 | 23.89 | 23.82 | 0.36 | 0.36 |
| 3 | 26.76 | 26.82 | 26.94 | 0.34 | 26.75 | 27.02 | 26.96 | 0.53 | 26.37 | 26.86 | 26.91 | 1.12 | 0.72 |
| 2 | 29.38 | 29.03 | 29.13 | 0.62 | 28.53 | 29.42 | 29.49 | 1.84 | 28.61 | 28.93 | 29.11 | 0.88 | 1.17 |
| NC | - | - | - | - | - | - | - | - | - | - | - | - | - |
|  |  |  |  |  |  |  |  |  |  |  |  |  |  |
| PToV | | | | | | | | | | | | | |
| Log Quantity | Group 1 | | | | Group 2 | | | | Group 3 | | | | CV % |
|  | rep1 | rep2 | rep3 | CV % | rep1 | rep2 | rep3 | CV % | rep1 | rep2 | rep3 | CV % |  |
| 7 | 14.18 | 14.14 | 14.20 | 0.22 | 14.27 | 14.23 | 14.26 | 0.15 | 14.37 | 14.27 | 14.31 | 0.35 | 0.49 |
| 6 | 17.46 | 17.54 | 17.75 | 0.85 | 17.68 | 17.58 | 17.61 | 0.29 | 17.59 | 17.66 | 17.63 | 0.20 | 0.48 |
| 5 | 20.78 | 20.84 | 20.90 | 0.29 | 20.94 | 20.91 | 20.93 | 0.07 | 21.07 | 21.06 | 21.02 | 0.13 | 0.47 |
| 4 | 24.10 | 24.07 | 24.10 | 0.07 | 24.28 | 24.27 | 24.28 | 0.02 | 24.19 | 24.33 | 24.23 | 0.30 | 0.39 |
| 3 | 27.42 | 27.30 | 27.49 | 0.35 | 27.42 | 27.52 | 27.33 | 0.35 | 26.87 | 27.37 | 27.52 | 1.25 | 0.73 |
| 2 | 30.55 | 29.83 | 29.98 | 1.26 | 29.08 | 29.91 | 30.03 | 1.74 | 29.55 | 29.60 | 29.76 | 0.37 | 1.34 |
| NC | - | - | - | - | - | - | - | - | - | - | - | - | - |
|  |  |  |  |  |  |  |  |  |  |  |  |  |  |
| SADS-CoV | | | | | | | | | | | | | |
| Log Quantity | Group 1 | | | | Group 2 | | | | Group 3 | | | | CV % |
|  | rep1 | rep2 | rep3 | CV % | rep1 | rep2 | rep3 | CV % | rep1 | rep2 | rep3 | CV % |  |
| 7 | 14.61 | 14.63 | 14.62 | 0.07 | 14.69 | 14.59 | 14.64 | 0.34 | 14.85 | 14.74 | 14.77 | 0.38 | 0.60 |
| 6 | 18.09 | 18.11 | - | 0.08 | 18.17 | 18.11 | 18.17 | 0.19 | 18.17 | 18.19 | 18.25 | 0.23 | 0.29 |
| 5 | 21.50 | 21.52 | 21.63 | 0.32 | 21.63 | 21.54 | 21.51 | 0.29 | 21.71 | 21.71 | 21.72 | 0.03 | 0.43 |
| 4 | 24.99 | 24.91 | 24.96 | 0.16 | 25.14 | 25.16 | 25.16 | 0.05 | 25.07 | 25.19 | 25.14 | 0.24 | 0.41 |
| 3 | 28.10 | 28.11 | 28.15 | 0.09 | 28.21 | 28.41 | 28.28 | 0.36 | 27.96 | 28.16 | 28.25 | 0.53 | 0.45 |
| 2 | 30.92 | 31.51 | 31.61 | 1.19 | 30.54 | 32.54 | 32.08 | 3.30 | 30.66 | 32.15 | 32.31 | 2.87 | 2.35 |
| NC | - | - | - | - | - | - | - | - | - | - | - | - | - |

Rep：replicate; “-”: Cq>35 or no singal detected, negative (-); Group 2 and Group 3 were the same detection assay as Group 1, which were performed with a time interval of 7 days. “NC” means taking the same volume of nuclease-free water as the template.

Based on the Cq values, we calculated the standard equation, R^2^ and E value with software GraphPad Prism 8: PEDV: Group 1: Y = -3.211*X+37.44, R^2^=0.9993, E=104.85%; Group 2: Y = -3.173*X+37.24, R^2^=0.9972, E=106.61%; Group 3: Y = -3.107*X+36.91, R^2^=0.9972, E=109.82%. PDCoV: Group 1: Y = -3.131*X+35.94, R^2^=0.9961, E=108.63%; Group 2: Y = -3.194*X+36.15, R^2^=0.9951, E=105.63%; Group 3: Y = -3.118*X+35.78, R^2^=0.9936, E=109.28%. PToV: Group 1: Y = -3.213*X+36.83, R^2^=0.9990, E=104.76%; Group 2: Y = -3.139*X+36.49, R^2^=0.9964, E=108.24%; Group 3: Y = -3.105*X+36.33, R^2^=0.9968, E=109.92%. SADS-CoV: Group 1: Y = -3.346*X+38.17, R^2^=0.9996, E=99.01%; Group 2: Y = -3.413*X+38.61, R^2^=0.9997, E=96.33%; Group 3: Y = -3.365*X+38.42, R^2^=0.9995, E=98.23%.

**Table S6** Information of clinical samples.

| Batch | Number of samples | Information of samples | Initial identification method | Results | Conformity rate between the two methods |
| --- | --- | --- | --- | --- | --- |
| 1 | 45 | Recently collected (December 2019) | Singleplex conventional PCR & DNA sequencing | Only PEDV*9 | 100 % |
| 2 | 56 | Previously collected (2017-2019) | Singleplex conventional PCR & DNA sequencing | PEDV*31, PDCoV*16, SADS-CoV*1, PToV*3, (PEDV+PToV)*4, (PEDV+PToV+PDCoV)*1 | 100 % |
